# Stem cell donor registry activities during the COVID-19 pandemic: a field report by DKMS

**DOI:** 10.1038/s41409-020-01138-0

**Published:** 2020-11-20

**Authors:** Thilo Mengling, Gabi Rall, Stefanie N. Bernas, Nadia Astreou, Sandra Bochert, Torben Boelk, Deborah Buk, Konstanze Burkard, Dennis Endert, Katrin Gnant, Sabine Hildebrand, Hatice Köksaldi, Isabelle Petit, Jürgen Sauter, Susanne Seitz, Julia Stolze, Karin Weber, Maren Weber, Vinzenz Lange, Julia Pingel, Alexander Platz, Thomas Schäfer, Johannes Schetelig, Edith Wienand, Sirko Geist, Elke Neujahr, Alexander H. Schmidt

**Affiliations:** 1 DKMS, Tübingen, Germany; 2 DKMS Life Science Lab, Dresden, Germany; 3 DKMS Registry, Tübingen, Germany; 4 DKMS Stem Cell Bank, Dresden, Germany; 5 DKMS, Clinical Trials Unit, Dresden, Germany; 6 Medizinische Klinik I, University Hospital Carl Gustav Carus, Dresden, Germany

**Keywords:** Bone marrow transplantation, Haematopoietic stem cells

## Abstract

The COVID-19 pandemic has serious implications also for patients with other diseases. Here, we describe the effects of the pandemic on unrelated hematopoietic stem cell donation and transplantation from the perspective of DKMS, a large international donor registry. Especially, we cover the development of PBSC and bone marrow collection figures, donor management including Health and Availability Check (HAC), transport and cryopreservation of stem cell products, donor recruitment and business continuity measures. The total number of stem cell products provided declined by around 15% during the crisis with a particularly strong decrease in bone marrow products. We modified donor management processes to ensure donor and product safety. HAC instead of confirmatory typing was helpful especially in countries with strict lockdowns. New transport modes were developed so that stem cell products could be safely delivered despite COVID-19-related travel restrictions. Cryopreservation of stem cell products became the new temporary standard during the pandemic to minimize risks related to transport logistics and donor availability. However, many products from unrelated donors will never be transfused. DKMS discontinued public offline donor recruitment, leading to a 40% decline in new donors during the crisis. Most DKMS employees worked from home to ensure business continuity during the crisis.

## Introduction

The ongoing 2019/20 COVID-19 pandemic [1, 2] also affects the treatment of non-COVID-19 patients as it places significant stress on national health systems and societies as a whole. In addition, many people in need of treatment avoid visiting health care facilities due to fear of infection [3, 4]. In this article, we examine the effects of the pandemic on unrelated hematopoietic stem cell donation and transplantation from the stem cell donor registry perspective using the example of DKMS. DKMS is a large donor registry with more than 10 million donors from six countries (Germany, Poland, United States, United Kingdom, Chile, India) and has so far facilitated over 85,000 stem cell collections. In 2018, the DKMS share of all unrelated stem cell donations worldwide was 39.5% [5]. In this report we present global and country-specific DKMS figures. With regard to the description of challenges and activities, we focus on DKMS Germany, by far the largest DKMS country organization (>6.6 million registered donors, >72,000 donations facilitated), unless otherwise explicitly stated. There are only minor differences to the other DKMS entities as the various country organizations have closely coordinated their efforts to cope with the COVID-19 crisis.

### Stem cell collections

**Fig. 1:**
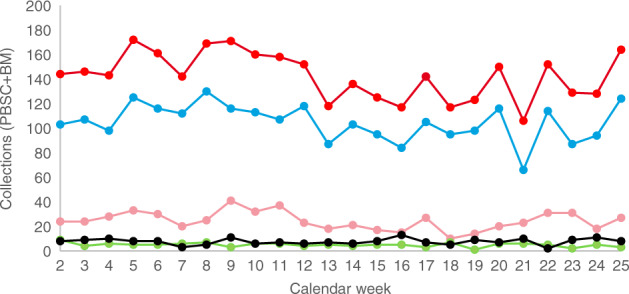
Collections (bone marrow (BM) and peripheral blood stem cells (PBSC)) of DKMS donors by donor country by calendar week. Red: DKMS total; blue: DKMS Germany; pink: DKMS Poland; black: DKMS US; green: DKMS UK. DKMS Chile and DKMS-BMST India are not displayed but included in the total figures.

During the COVID-19 pandemic, stem cell collections (peripheral blood stem cells and bone marrow) of DKMS donors decreased by 15.9% worldwide from 156.2/week (calendar weeks (CW) 2–12) to 131.3/week (CW 13–25; Fig. 1; Table 1). Corresponding values for DKMS Germany donors were 113.2/week (CW 2–12) and 97.5/week (CW 13–25), indicating a 13.8% decrease. Even though the beginning of the crisis was somewhat arbitrarily defined for this analysis, it reflects the significant drop in collections from CW 12 (March 16–22) to CW 13 (March 23–29). The World Health Organization declared the COVID-19 outbreak a pandemic on March 11 [6]. In order to reconcile the chronological developments of the figures presented here with general developments during the COVID-19 pandemic, comprehensive timelines [7, 8] may be used. A DKMS-specific timeline of events and decisions related to the pandemic is given in Table 2.

**Table 1:** Mean weekly collections of DKMS donors by donor country before and during the crisis.

DKMS entity/donor country	Calendar weeks 2–12	Calendar weeks 13–25	Change in %
	(Jan 6–Mar 22)	(Mar 23–June 21)	
Germany	113.2	97.5	−13.8
Poland	28.8	20.9	−27.4
United States	7.4	7.8	6.6
United Kingdom	5.5	4.4	−20.9
Chile	0.6	0.6	−3.3
India	0.6	0	−100
Total	156.2	131.3	−15.9

**Table 2:** DKMS-specific timeline of events and decisions during the COVID-19 pandemic.

Date (calendar week)	Events/decisions
January (1–5)	• Start update pandemic plan • Check responsibilities during pandemic
February 26 (9)	• Start monitoring if donor recruitment drives are still feasible • Check IT resources required to enable most teams to work from home • Check stocks of surgical masks and disinfectants • Prepare donor eligibility criteria and statements on donor safety
February 28 (9)	• Pandemic coordination group installed • Prepare protective measures in the offices • Expand working from home
March 2 (10)	• Decision: No donor recruitment drives in risk areas • Procure additional IT equipment for working from home
March 9 (11)	• Decision: Employees belonging to vulnerable groups should work from home • Decision: DKMS US office in New York will be closed (still closed as of September 2020)
March 12 (11)	• Last DKMS donor recruitment drive in Germany
March 15 (11)	• Last DKMS donor recruitment drive globally (United States) • First confirmed SARS-CoV-2 infection of a DKMS employee
March 16 (12)	• DKMS main office in Tübingen closed (partially re-opened from May 11)
March 20 (12)	• DKMS offers Health and Availability Check instead of conventional confirmatory typing
March 16–20 (12)	• Massive peak in postponements of workup requests
March 24 (13)	• New DKMS policy on cryopreservation: - General acceptance of cryopreservation requests due to COVID-19 concerns - No SARS-CoV-2 testing unless for donor reasons - Recommend to consider switch from bone marrow to PBSC wherever possible
March 26 (13)	• “Capacity Board” established
March 30 (14)	• DKMS newsletter to transplant centers: Information on reduced capacity for bone marrow and donor lymphocyte collections
April 6 (15)	• “Cockpit cargo” process as standard for stem cell product transports between Germany and the United States
April 7 (15)	• DKMS newsletter to transplant centers: Higher cell count requests accepted due to need for cryopreservation, further COVID-19-related process changes
April 14 (16)	• First reports of stem cell products that will not be infused • Start tracking of non-infused products
April 16 (16)	• Decision: Refuse unrealistic workup requests for cryopreserved products (i.e., requests where it is foreseeable that cell count will not be sufficient after cryopreservation and thawing) to protect donors from futile collections
July 8 (28)	• Revised DKMS policy on cryopreservation (incorporated the recommendations from WMDA SEAR Rapid Alert 07–2020 [ref. 27]: - Recommendation to use fresh products where transport and donor availability are safe - Bone marrow only if excess cell counts are expected - Transplantation as soon as feasible after arrival of the product - Thorough assessment of recipient’s eligibility for immediate transplantation before scheduled start of donation

**Fig. 2:**
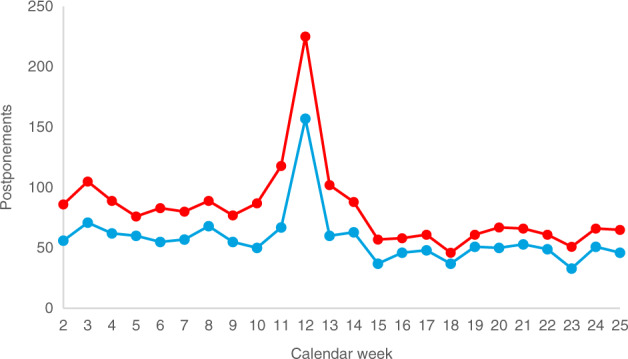
Postponements of workup requests by calendar week. Red: DKMS total; blue: DKMS Germany.

The decline in collections from CW 13 on was partly due to a large number of postponement requests for ongoing donor workups in CW 12 (Fig. 2). These requests were likely to be in response to a rapid worsening of the crisis in mid-March. On March 17, the German Foreign Ministry issued a worldwide travel warning for all tourist travel abroad for the first time in its history [9]. Many travel restrictions and border closings worldwide were put into effect around the same time [10].

**Fig. 3:**
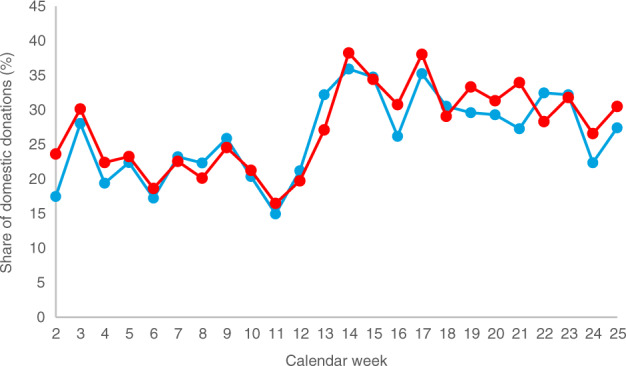
Share of domestic donations by calendar week. Red: DKMS total; blue: DKMS Germany.

During the crisis, we observed a stronger preference for domestic donors than usual. The share of domestic donations from all DKMS donors increased from 22.0% prior to the crisis (CW 2–12) to 31.8% during the crisis (CW 13–25; Fig. 3). The corresponding values for donations from DKMS Germany donors were almost identical, namely 21.2% (CW 2–12) and 30.5% (CW 13–25). The reason for this observation is most probably due to actual or presumed simpler logistics associated with a domestic donation in the crisis situation (see paragraph *Transport of stem cell products*).

Regarding recipient countries, we observed a 16.2% increase in the number of donations for German patients from 29.7/week (CW 2–12) to 34.5/week (CW 13–25), while it was otherwise generally declining (Table 3). On the basis of these figures, it remains speculative whether the reason for this discrepancy is that Germany has so far been comparatively only mildly affected by the pandemic [11, 12]. It is also possible that the discrepancy is merely the result of a combination of the very large DKMS Germany donor file and the general preference for domestic stem cell products.

**Table 3:** Mean weekly collections of DKMS donors (all country organizations) by recipient country before and during the crisis.

Recipient country	Calendar weeks 2–12	Calendar weeks 13–25	Change in %
	(Jan 6–Mar 22)	(Mar 23–June 21)	
United States	39.7	33.8	−14.8
Germany	29.7	34.5	16.2
United Kingdom	11.1	7.9	−28.6
France	10.0	8.1	−19.2
Italy	8.2	5.7	−30.4
Poland	7.1	6.2	−12.1
ROW	50.4	35.0	−30.5
Total	156.2	131.3	−15.9

At DKMS Germany, donation numbers were not significantly affected by COVID-19-related donor non-availability: Of all 330 donors who were not available in the workup process in the crisis (start date: March 16; data retrieval date: June 15), only 7 (2.1%) were due to COVID-related reasons (Table 4). In contrast, other DKMS country organizations reported an increase in donor non-availability at the workup level; not due to more COVID infections, but donor concerns and public health measures to shield vulnerable persons. Interestingly, donor non-availability at confirmatory typing (CT) level decreased significantly from 22.6% (CW 2–12) to 13.4% (CW 13–25) at DKMS Germany. Other DKMS country organizations showed similar results. Possible explanations could be that requested donors had fewer appointments, were at home more often, and had more time due to the crisis.

**Table 4:** Reasons for COVID-19-related donor non-availability at workup level (DKMS Germany, start date: March 16; data retrieval date: June 15).

COVID-19-related reason for donor non-availability	*N*
Donor concerns about COVID-19	1
SARS-CoV-2 infection confirmed	1
Suspected SARS-CoV-2 infection, not confirmed	2
Risk contact, travel history	3
Total	7

**Fig. 4:**
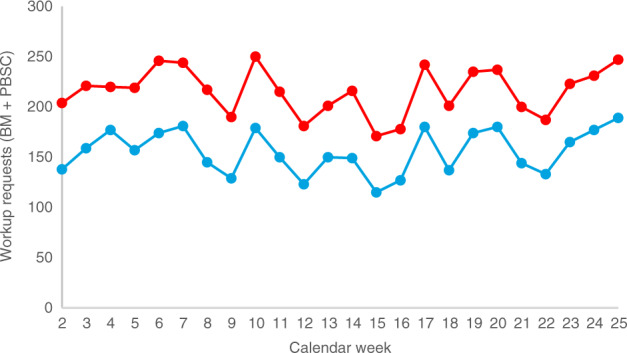
Workup requests (bone barrow (BM) and peripheral blood stem cells (PBSC)) by calendar week. Red: DKMS total; blue: DKMS Germany.

The current trend (CW 23–25) in the number of workup requests (DKMS Germany: 177 requests/week; DKMS total: 234 requests/week; Fig. 4) suggests a recovery in collection numbers in the near future.

### Bone marrow collections

Two aspects complicate bone marrow collections during the COVID-19 crisis: Firstly, bone marrow collection capacities were uncertain due to staff reasons or COVID-19-related changes in the allocation of resources in the CC. Specifically, three of the eleven bone marrow collection facilities cooperating with DKMS Germany have temporarily stopped performing bone marrow collections in the crisis, another two provided only reduced capacities. Secondly, cell loss due to cryopreservation frequently performed during the crisis (see section *Cryopreservation of stem cell products*) is particularly problematic because low cell counts are a common problem in bone marrow collections anyway [13]. Therefore, we informed the transplant centers on March 30 that bone marrow requests would only be accepted without explicit clarification if there was an obvious reason why bone marrow was preferred (pediatric patient, nonmalignant disease). In the same communication, we also emphasized that the CC might not clear the donor for bone marrow donation if the expected viable cell content after cryopreservation and thawing is below the threshold for a promising transplantation (e.g., due to an unfavorable donor-patient weight ratio). As a result of the COVID-19 crisis, the proportion of bone marrow donations from DKMS Germany donors has decreased from 16.5% (CW 2–12) to 7.6% (CW 13–25). The fact that typical indications for bone marrow do not require an immediate transplantation might have further reduced the number of marrow collections.

### Donor management

Donor management at the work-up level and in the context of physical examination and donation required numerous adjustments, such as:

- On March 17, 2020 we implemented an additional health questionnaire (including questions regarding residing in risk areas or contact with persons exposed to SARS-CoV-2) which is taken at the initial information session with the donor.
- Public transportation, which is the usual means of transportation to and from the CC was switched to private cars, taxis and driving services in order to minimize the risk of infection.
- Our cooperating CC took further safety measures, such as telephone interviews with donors prior to the physical examination or donation, compulsory masks, or fever measurements before entering the facility. Furthermore, unlike normal practice, no additional persons were allowed to accompany donors in the CC.
- We made agreements with hotels making sure that our donors belonged to the restricted group of people who were still allowed to check into hotels. (Temporarily only very few hotels were still open for pilots, train drivers, police officers and other such individuals working in the public sector.)

Donors were tested for SARS-CoV-2 infection according to the requirements of the competent authorities or where there was reason to suspect the donor could be infectious regardless of the donation, for example due to COVID-typical symptoms, contact with an infected person or a stay in a risk area. In Germany, the competent authority for blood and blood products does not recommend screening healthy donors for SARS-CoV-2 [14]. We do not regard a product quarantine until a SARS-CoV-2 infection has been ruled out as advisable, especially since it is considered unlikely that SARS-CoV-2 is transmitted by blood products [15]. In accordance with the recommendations of the European Society for Blood and Marrow Transplantation (EBMT) [16] and the World Marrow Donor Association (WMDA) [17] we exclude donors with SARS-CoV-2 infection from stem cell donation until 28 days after recovery, although exceptions are possible in urgent cases. Thus far, there has only been one case at DKMS Germany where a confirmed SARS-CoV-2 infection prevented a stem cell donation after final clearance (Table 4). The fact that recommendations were quickly aligned between relevant international organizations such as EBMT, WMDA or the National Marrow Donor Program (NMDP) of the United States substantially facilitated donor management during the crisis.

### Health and availability check

Very few errors occur when modern HLA typing methods such as next generation sequencing are applied in accredited laboratories. Therefore, it seems questionable if the standard execution of a confirmatory typing (CT)—a process that has been established when serological HLA typing was state-of-the-art—before each donor workup is really useful. We concluded to introducing the Health and Availability Check (HAC) as an alternative to CT [18]. HAC includes providing detailed information on the two ways of donation to the donor via phone, checking if the donor is still interested and available to donate, and screening the donor’s health state using the common CT health questionnaire. No blood samples are taken for confirmatory typing or the determination of infectious disease markers. These analyses are only done during donor workup. Since only about every fourth donor with a CT request gets into donor workup [18], we are convinced that HAC has the potential to reduce donor burden as well as search costs and duration. Clearly, the search coordinator has the final say on whether to request an HAC or a standard CT. In the COVID-19 crisis, two further strong arguments for HAC emerged: Firstly, in order to protect the donor from a COVID-19 exposure, it is advisable to avoid, if possible, taking a blood sample, which is usually done by a general practitioner. Secondly, logistical problems may arise when transporting the CT blood sample. These arguments prompted us to send a newsletter to TC on March 23, urging them to request HAC instead of CT. We offer the processing of HAC requests free of charge. During the crisis, HAC proves to be of special value in countries with strict lockdowns as, for example, the UK. Accordingly, DKMS UK performed HAC instead of CT in 96.4% of cases during the lockdown (CW 13–22).

### Transport of stem cell products

When the crisis worsened in mid-March with border closures, travel bans, quarantine requirements for couriers and massive cancellations of passenger flights, it quickly became clear that the safe transport of stem cell products to transplant centers (TC) would be one of the biggest challenges for stem cell donor registries. Fortunately, key institutions quickly recognized these difficulties and took appropriate measures. For example, the European Commission clarified that Substances of Human Origin including hematopoietic stem cells are considered critical goods for which free circulation within the European Union is crucial [19, 20]. For the United States, the NMDP Patient Advocacy group managed to receive a blanket travel ban waiver from the Centers for Disease Control and Prevention, thus ensuring that European couriers could transport stem cell products into the United States despite the travel ban [21]. From our perspective, it is no exaggeration to say that these difficult days were also a shining hour of international cooperation, both within the stem cell community and far beyond.

In response to the logistical challenges of the COVID-19 crisis, two new transport modes have emerged: The first new process involves the transport of stem cell products in the cockpit or crew compartment of cargo aircraft under the supervision of the pilot or co-pilot. This process has the benefit that no actual stem cell courier is required, thus avoiding complications caused by travel bans and quarantine requirements. Furthermore, it takes advantage of the fact that cargo air traffic has been affected much less than passenger air traffic which at times has come to an almost complete standstill. The “cockpit cargo” process is currently supported by Lufthansa and Latam. By August 10, 453 stem cell products from DKMS Germany donors were transported this way to the USA, Turkey, Argentina, Chile, and India. Since cargo planes fly with a lower frequency than passenger aircraft have before the crisis, the cockpit cargo process may result in longer transport times than usual. However, this process turned out to be the by far best possible solution for many routes during the crisis.

The second new process includes cryopreservation of the stem cell product at or near the CC and subsequent transport as airfreight in a dry shipper, in accordance with the transport of umbilical cord blood units. In addition to the same advantages as in the cockpit cargo process (no courier required, sufficient cargo air traffic), this process is relevant for particularly complex transports, e.g., from Germany to Australia or South Africa, since a cryopreserved product can be kept in the dry shipper for up to about 10 days. So far (collections until June 29), 22 stem cell products from DKMS Germany donors have been transported this way. Three of these products were damaged during transport, one of them so severely that it had to be discarded. The reasons for these incidents are still under investigation.

Besides these two new temporary standard processes, numerous creative individual case solutions were required such as diplomatic flights/cars, product handovers at closed borders and transports in ambulances.

### Cryopreservation of stem cell products

From mid-March onward, many transplant physicians could—given the imponderables of the crisis—only start conditioning a patient once the corresponding stem cell product had safely arrived at the clinic. This approach, which was also recommended, e.g., by the EBMT [16] and the European Centre for Disease Prevention and Control “in situations where there is an increased risk that a donor would become unavailable at the time of planned transplantation due to community-acquired COVID-19, travel restrictions or logistical difficulties” [22], requires the cryopreservation of the product at the TC. Furthermore, in some cases cryopreservation at or near the CC is necessary for reasons of transport logistics (see section *Transport of stem cell products)*. Accordingly, during the crisis, we have extensively approved cryopreservation requests, although we have called for a start of conditioning as soon as possible, at least within 7–14 days after arrival of the cells. This is contrary to our normal approach, as we otherwise handle cryopreservation requests for stem cell products from DKMS donors very restrictively in order to avoid that collected products from unrelated donors are ultimately not transfused.

Of all stem cell products collected from DKMS Germany donors from March 1 to August 15, 2020, 1736 were cryopreserved. According to the information currently available to us, 35 of these 1736 stem cell products (2.0%) will definitely or very likely not be transfused (data retrieval date: August 19, 2020). The reasons for non-transplantation include patient death, deterioration of patient status, and dissatisfaction with product specifications (cell count, viability) before cryopreservation or after thawing. For another 87 collected and currently cryopreserved stem cell products (5.0%), we lack up-to-date infusion plans or have not received a statement from the TC at all. We expect that several of these products will not be transfused either. Furthermore, confirmation of product transfusion is still missing for 197 cases of which 107 products (6.2% of all cryopreserved products) should have been transfused by August 18. Taken together, we are concerned that about 5–10% of the cryopreserved products might ultimately not be transfused [23].

In our opinion, these data suggest that cryopreservation of a stem cell product from an unrelated donor should only be performed in exceptional cases. Stem cell harvest from an unrelated donor without subsequent transfusion of the product must be avoided for donor safety and ethical reasons if at all possible. Without any reasonable doubt, such an exceptional situation prevailed in mid-March and the following weeks. The question arises, however, whether or in which cases cryopreservation is still justified since transport of stem cell products is again safely possible between many places. We are concerned the large number of products not infused might have a negative impact on future willingness for volunteer donation.

We therefore think that the decision on cryopreservation of stem cell products from unrelated donors should be made on a case-by-case basis as the crisis progresses. Possible criteria that speak against cryopreservation at the TC are stable transport connections between CC and TC, long transport duration resulting in suboptimal viability upon arrival at the TC, low infection rate at the donor’s place of residence, high donor availability, bone marrow transplantation, and unfavorable donor/patient weight ratio.

Concerning the management of non-transplanted products, it would be desirable to keep them cryopreserved and make them available to other patients via a listing in the stem cell donor registries or at least to save them as potential autologous products for the donors. However, the regulatory hurdles are very high, especially for the first option. Currently, we are striving to prevent the products from being discarded and to keep them cryopreserved either in the transplant center or in the DKMS Stem Cell Bank. In some cases, however, we receive the information that the transplant will not take place only after the product has been discarded. We are aiming at a solution that on the one hand considers product safety, on the other hand takes into consideration the exceptional COVID-19-related situation and the altruistic commitment of unrelated donors.

### Donor recruitment

**Fig. 5:**
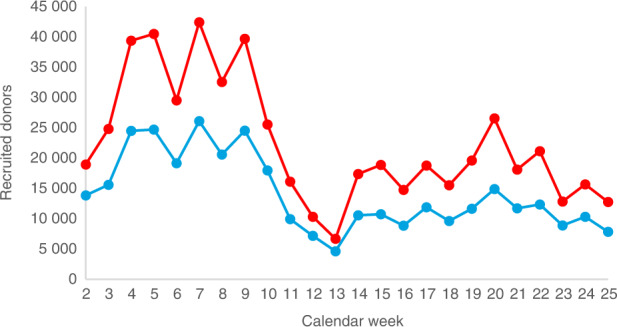
Recruited donors (start of data processing at DKMS) by calendar week. Red: DKMS total; blue: DKMS Germany.

Compared to ensuring the treatment of patients with potentially life-saving stem cell products, donor recruitment during the COVID-19 crisis is certainly of secondary importance. Furthermore, the execution of donor drives would be neither reasonable nor permitted in the countries where DKMS recruits new donors. DKMS has therefore stopped all public offline donor recruitment activities until further notice. The so far last DKMS donor drive in Germany took place on March 12, the last DKMS donor drive altogether on March 15 in the United States. On the contrary, there is no reason to stop online donor recruitment activities as they do not involve any infection risk: Interested individuals register online via the DKMS website, receive a buccal swab set with consent form, apply the swabs at home and send them together with the signed consent form by mail to the HLA laboratory. As a result of this temporary policy, the number of newly registered donors at DKMS Germany has decreased considerably by 38.1% in the crisis, from 10,516 donors/week (CW 2–12) to 6511 donors/week (CW 13–25). The corresponding global DKMS figures are 18,561 (CW 2–12) and 10 312 (CW 13–25; -44.4%; Fig. 5).

The samples of all new DKMS donors are genotyped at the DKMS Life Science Lab (Dresden, Germany). The newly available resources resulting from the decrease in new donors were used, among other things, to support the University Hospital Dresden (UKD) in SARS-CoV-2 testing by isolating the viral RNA from throat swabs. This cooperation increased the daily capacity of the UKD from 300 to 900 tests [24].

### Business continuity

Pandemic-related activities of DKMS are coordinated by a pandemic group (Head: TM). The group was established in 2010 in response to the H1N1 pandemic and at that time developed a pandemic plan. Although the group had hardly been active since then, it was able to use the preparatory work and organizational structures after its revitalization in January 2020. The pandemic group regularly evaluates the current situation and recommendations by German authorities and other relevant organizations in order to develop guidelines for DKMS employees. The aim is to reduce the risk exposure of employees and, in the event of an employee becoming infected, to minimize the need for contact tracing and extensive quarantine measures.

Therefore, an almost complete transfer of activities from the office to working from home was enabled within a very short time. For this purpose, hardware and software licenses had to be procured, processes adapted and employees trained accordingly to comply with, e.g., IT security and data privacy rules in this new setting. As a result, at the DKMS headquarters in Tübingen, the share of days working from home rose from 12.5% in February via 53.2% in March to 87.9% in April.

Another challenge is that the COVID-19 crisis changed the workload of the various DKMS departments in opposite ways. For example, despite the slight decrease in workup requests, the total workload increased considerably in the Workup department due to the crisis-related complex transport and cryopreservation issues while it decreased in the Donor Recruitment department due to the stop of public offline donor recruitment activities. For this reason, a virtual marketplace (“Capacity Board”) has been set up on the DKMS intranet where employees with free resources and their specific competencies and understaffed tasks are matched.

### Summary and perspectives

The 2019/20 COVID-19 pandemic is the most significant health-related phenomenon for many decades, with numerous implications for societies in general and national health systems in particular. The collection figures presented in this paper suggest that effects of the pandemic on unrelated stem cell transplantation are clearly present but still moderate. Since donations from DKMS donors account for about 40% of all unrelated stem cell donations worldwide [5], this assessment should reflect the situation on a global scale. There are no indications that the collection figures will continue to fall in the near future. On the contrary, the noticeable recent increase in the number of workup requests suggests that the number of donations will recover in the short term. Longer-term forecasts are of course hardly possible due to the unpredictable course of the pandemic.

In the early phase of the pandemic (mid-March and the weeks following), ensuring the safe transport of stem cell products was probably the most important task of donor registries. After that challenge had largely been overcome, problems have come to the fore in connection with the widespread cryopreservation of stem cell products. In our view, numerous non-transfused products underline that it is necessary to return to the pre-crisis standard of using fresh products as soon as possible. In contrast to widespread cryopreservation, we believe it would be desirable for HAC to continue to be widely used after the crisis because in our opinion it is a clear process improvement [18].

The association of immunogenetic parameters with the severity of COVID-19 disease course is a scientific question of high practical relevance. Interestingly, donor registries are in a very good position to conduct such studies quickly and cost-efficiently as they administer large files of donor immunogenetic data. The particularly broad standard typing profile of new DKMS donors [25, 26] is also very helpful in this task. DKMS is currently conducting an e-mail-based survey of around 4.5 million registered donors in Germany on a possible SARS-CoV-2 infection they may have experienced, in order to correlate these data with existing immunogenetic donor registry data. To our knowledge, various stem cell donor registries are planning or preparing similar studies.

In summary, it seems justified to state that—not least due to the successful collaboration of the “stem cell community”, i.e., donor registries, WMDA, CC and TC, professional societies as EBMT, and courier companies—it has largely been possible to protect patients and donors in difficult times.
